# Symptom Endorsement and Sociodemographic Correlates of Postnatal Distress in Three Low Income Countries

**DOI:** 10.1155/2016/1823836

**Published:** 2016-02-15

**Authors:** Amanda J. Nguyen, Emily E. Haroz, Tamar Mendelson, Judith Bass

**Affiliations:** Department of Mental Health, Johns Hopkins School of Public Health, 624 North Broadway, 8th Floor, Baltimore, MD 21205, USA

## Abstract

*Background*. Maternal mental illness has been implicated in adverse child development outcomes. Factors such as context and culture may influence experiences of maternal distress and explain differences in outcomes across settings.* Methods.* We analyzed baseline data from 5,647 mothers in Ethiopia, India (Andhra Pradesh), and Vietnam participating in an ongoing cohort study (Young Lives) to compare symptom endorsement and sociodemographic correlates of distress. Maternal distress was assessed using the Self-Reporting Questionnaire-20 Items (cutoff: ≥8). Logistic regressions were stratified by sample to identify correlates of distress.* Results.* Symptom endorsement was similar among distressed women, particularly with regard to feeling unhappy (76%, 80%, and 79%). Notable differences were observed in three items assessing Depressive Thoughts, which were most highly endorsed in Ethiopia (49%–56%). Having a child experiencing a life-threatening event was correlated with distress in all three samples. A variety of correlates were unique to only one sample.* Conclusions.* There were multiple similarities but also notable differences across sites in the expression and correlates of maternal distress. Feeling unhappy appears to be a hallmark feature of distress. Correlates highlight the relationship between distress and indicators of poverty, child wellbeing, and economic shocks. Differences demonstrate the value of further exploration of cross-cultural differences.

## 1. Background

Depressive and anxiety disorders are a leading contributor to global disability, accounting for more than half of the total neuropsychiatric burden of disease [1]. These disorders present a higher burden among women than men with particular implications for women of childbearing age, for whom depression is the number one cause of disease burden in both high and low resource settings [2]. A systematic review of studies from low and middle income countries (LMIC) reported a weighted mean depression prevalence of 19.8% in the postnatal period, indicating that the burden may be higher in low resource as compared with high-resource settings [3]. Additionally, studies suggest women may be at higher risk for anxiety disorders during the perinatal period [4]. Although less research has focused on perinatal anxiety or broader categories of distress, focusing on depression alone is problematic because symptoms of depression and anxiety often cooccur [5, 6]. Therefore, in the present study we examine a broader construct of maternal distress, defined as elevated depressive, anxious, and somatic symptoms within the perinatal period. These mental health problems cause significant role impairment and have been implicated in a range of negative child development outcomes [7–9] such as problems related to infant growth and cognitive development [10, 11], greater number of diarrheal episodes and poorer adherence with recommended immunization schedules [12], and heightened risk of insecure attachment and child behavioral problems [5, 13].

Culture can influence the expression of distress and other mental health problems and must be considered when studying mental health cross-culturally [14]. There is growing recognition that symptom presentation of common mental disorders may vary across individuals and groups, which results in diverse clinical presentations and potential for different risk factors and responses to treatment [15–19]. For example, findings that appetite changes are not reliably associated with perinatal depression [20] and that sadness presents as less of a core feature in late life depression [21] suggest improved accuracy of the identification of these types of problems among various subgroups. The prevalence of specific depressive and anxiety symptoms has also been shown to vary across cultures [16, 22, 23], and in certain populations standardized screening instruments have been found to be less informative than indigenous measures for capturing common mental health problems [24, 25]. Likewise, researchers have examined the impacts of potential cultural differences in somatization on depression case finding and outcomes [26–28]. However, exploration of culture's impact on more general maternal distress has been underexplored in the literature to date.

A number of social and demographic factors have also been found to vary by context in their association with increased risk for maternal common mental disorders (CMD) in LMIC [3, 7]. Fisher et al. [3] identified 47 studies across 17 countries on depression either during pregnancy or in the year following birth. Reported risk factors included social and economic circumstances (poverty, economic hardship, young age, being an ethnic minority, and being unmarried), family and relationship stressors (intimate partner problems, family conflict, low social support, and having many children), pregnancy factors (unwanted or unintended pregnancy, poor maternal health during pregnancy, pregnancy complications, and past abortion or stillbirth), and child factors (having an infant with health problems, prolonged infant crying, experiencing the death of a child, and having a female child in certain cultures). Higher education, having employment, being in the ethnic majority, and receiving postpartum care were identified as protective factors.

The current study explores the experience of clinically significant levels of maternal distress symptoms across three LMIC settings, Ethiopia, India (Andhra Pradesh), and Vietnam. These settings share challenges common to low income countries, such as high poverty and susceptibility to natural disasters, but also represent diverse cultural and sociodemographic perspectives. Ethiopia is an ethnically and linguistically diverse country where, in addition to the predominant Christian and Muslim religions, many people simultaneously adhere to indigenous belief systems [29, 30]. India, a majority Hindu country with Muslim and Christian minorities, has historically been characterized by a caste system that has influenced social norms and access to resources [31]. The people of Vietnam are predominantly Kinh ethnicity, with many small ethnic minority groups [29]. Family, education, and social structures in Vietnam have been influenced by the “three teachings” of Confucianism, Taoism, and Buddhism; a majority of the Vietnamese also report participating in ancestor worship [30]. With respect to sociodemographic indicators, in 2002 at the time of baseline data collection, the three countries differed in their levels of disadvantage, with Vietnam ranked highest in the United Nations Development Program (UNDP) Human Development Index (109) relative to India (124) and Ethiopia (168) [32]. Consistent with these rankings, Vietnam was characterized by longer life expectancy and higher rates of adult literacy and secondary school enrollment than the other two countries, with India ranking below Vietnam and Ethiopia reporting the poorest outcomes of the three countries in these domains [32]. The aims of this paper were to (1) compare endorsement of specific symptoms by mothers meeting criteria for maternal distress in these three settings and (2) evaluate the consistency of associations between maternal distress and recognized risk factors. These aims were exploratory, as the literature does not suggest clear predictions regarding how cultural and contextual differences across our samples will affect expression and correlates of maternal distress.

## 2. Methods

This cross-sectional, secondary analysis uses baseline data collected in Ethiopia, India (Andhra Pradesh), and Vietnam in 2002 as part of Young Lives (YL), an ongoing, longitudinal study of childhood poverty [33, 34]. Although YL is also following a Peruvian cohort, Peru was not included in this study as the mental health symptom-level data are not publicly available. The countries were selected for inclusion in the Young Lives study to reflect a diversity of experiences in LMIC [33]. At the time when baseline data were collected, Ethiopia was in the midst of a famine, was slipping back on progress made toward millennium development goals, and had high mortality, adult illiteracy, and child malnutrition. India was showing mixed progress toward development goals and also had high adult illiteracy and child malnutrition. By contrast, Vietnam had relatively lower levels of mortality, child malnutrition, and adult illiteracy [32]. This breadth of experience allows for an exploration of how context may affect experiences of maternal distress.

Data were collected in each country on approximately 2000 mothers of infant children using sentinel site surveillance. Detailed sampling methods and rationale have been described elsewhere [35]. Briefly, local experts identified 20 sites in each country to represent a range of geographic and socioeconomic environments, with oversampling of high poverty areas. For each site, lists of households with a child aged 6–18 months were compiled by observation and door-to-door surveys. One hundred index children and their caregivers were randomly sampled from each site to participate in the study.

The study team has published a detailed methods guide regarding research ethics and procedures [36]. Interviewers in all four sites were trained using a standard interviewer handbook (available at the UK Data Service [34]) that addressed ethics and consent, enrollment and interviewing procedures, and instructions for completion of each section of the common questionnaire. Training included role plays and practice sessions aimed at measuring the same items across the samples, while interviewer feedback during training was used to improve local translations [36]. Pilot studies were carried out in each country to evaluate the suitability of the questionnaire [37], and all interviews were completed in the local language of the primary caregiver. Informed consent was obtained for all adult participants, and the baseline survey obtained a response rate of over 90% in each of the three countries [37]. Ethical approval for the Young Lives study was granted by London School of Hygiene and Tropical Medicine [38]. While there were no exclusion criteria for the original data collection, this analysis excludes primary caregivers other than the biological mother (*n* = 108) and those missing mental health data (*n* = 255), resulting in 5647 cases: *n* = 1906, 1886, and 1855 in Ethiopia, Andhra Pradesh, and Vietnam, respectively.

### 2.1. Maternal Distress Caseness and Symptoms

**Figure 1:**
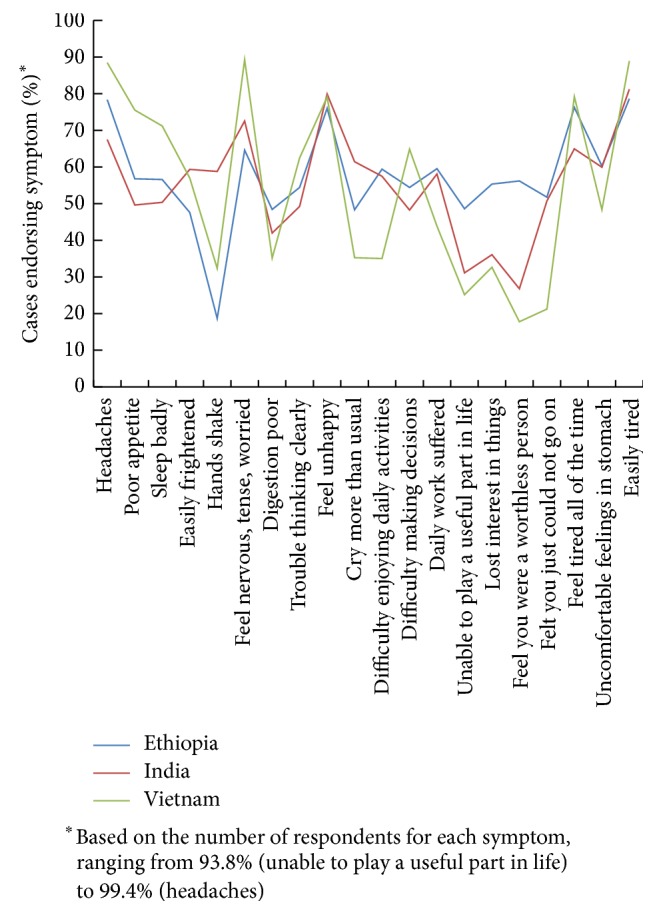
Prevalence of SRQ-20 symptom endorsement^*∗*^ in those identified as probable maternal CMD cases, by country.

A case of probable clinically relevant maternal distress was classified by the YL team using the Self-Reporting Questionnaire-20 Items (SRQ-20), a brief screening and case-finding (i.e., nondiagnostic) scale recommended by the World Health Organization for application in LMIC [39]. The SRQ-20 consists of 20 yes/no questions (no = 0; yes = 1) regarding somatic, depressive/anxious, and cognitive/energy symptoms experienced in the past 30 days (see Figure 1 *x*-axis for a list of all symptoms), summed to create a scale score ranging from 0 to 20 with higher scores indicating greater severity [40]. The scale has been translated and validated across several LMIC with sensitivity and specificity for psychiatric problems (as assessed in diagnostic interviews) ranging from 63 to 90% and 44 to 95%, respectively [39]. In India, cutoff scores of both 6 [41] and 8 or higher [42] have previously been used, although a more recent study reported an optimal cutoff of 12 or 13 among primary care attendees [43]. Early Ethiopian work identified optimal cutoffs of 8 or higher among clinic attendees and 5 or higher among nonclinic attendees [44], while a recent study using the SRQ specifically with perinatal women reported cutoffs of 3 or 7 and higher but noted study limitations in the ability to identify an optimal cutoff [45]. As the SRQ had not previously been validated in Vietnam, the English instrument was translated, back-translated, and validated with an optimal cutoff of 8 or higher [46]. Although the most efficient cutoff for determining caseness may vary by setting, a review of validation studies shows that a commonly used cut point is ≥ 8 for identifying a probable clinically significant case of maternal distress [40]. The YL dataset includes a binary case variable classifying women with probable distress by SRQ-20 scores below versus at or above 8 [37]. We also used the 20 symptom items to examine symptom endorsement among these samples.

### 2.2. Sociodemographic Correlates of Maternal Distress

Potential correlates of maternal distress were selected for analysis based on identification in prior research [3] and are listed in Table 1. Maternal factors include maternal age (in years), marital status, education level, and participation in livelihoods activities. Marital status was reported as married, single, divorced, or widowed but was recoded as binary (married/not married) for analysis due to low numbers of nonmarried women. Maternal education was defined on the basis of completion of primary school (yes/no). For livelihood, mothers reported the number of economic activities they participated in during the prior 12 months; this was coded as none (0), one (1), and two or more (2). Disability status was explored but excluded from the final analysis because the low number of disabled mothers precluded meaningful comparisons. Time lived in the community was also initially considered but excluded as it made no significant contribution to variance in the outcome across any of the three countries and was not supported by prior literature.

In addition to child age (months) and sex, pregnancy and child factors included whether or not the mother wanted the pregnancy (yes/no) and the child has a long-term health problem (recorded as the presence or absence of a long-term health problem) or has experienced a life-threatening event (recorded as whether or not the child had experienced a serious illness or injury where the mother believed the child might die). Birth weight was considered for inclusion but excluded due to missing data in approximately 50% of observations. Whether the mother had received antenatal care (none = 0; any = 1) was also explored but excluded from the final models due to lack of significant associations.

Household factors included community setting (urban/rural), number of children (dichotomized at 1–3 versus more than 3), having ever experienced the death of a child (yes/no), a household wealth index, and recent economic shocks. Household wealth index was a composite variable calculated by the YL team from a number of housing, consumption, and service factors, producing a continuous score ranging from 0 (lowest wealth) to 1 (highest wealth). Economic shocks were measured using a list of events the mother may have experienced since being pregnant with the index child, which negatively impacted household welfare (e.g., natural disaster, job loss, failed crops, severe illness or injury, migration, family death, and new educational expenses). These experiences were categorized as a binary variable indicating whether the household had experienced an economic shock (yes/no).

In light of previous research into the role of social capital in these same samples [47], a number of social capital variables (group membership, social support, citizenship, and cognitive social capital) were also considered. Group membership quantified the number of organizations the mother was a part of, analyzed as 0, 1-2, and 3 or more groups. Cognitive social capital (CSC) was a composite variable evaluating the mother's sense of community belonging, trust, and positive perception of others, analyzed as a low/medium (0) and high (1) CSC. Social support (the number of group, individual, and organizational sources from which the mother received social support in the past year, analyzed as 0, 1–4, and 5 or more sources) and citizenship activities (a binary none/some record of the mother's activities to join others in her community to enact change) were not included in the final models due to lack of significant contribution to variance.

### 2.3. Statistical Analysis

The analysis of individual symptom prevalence within the subset of those identified as probable cases (i.e., those with SRQ-20 scores at or above 8) was calculated by country using cross-tabulation. Chi-square analyses were used to evaluate differences in symptom prevalence across countries. Missing data were excluded for the symptom analysis, so that the proportion of endorsement for all symptoms was calculated based on the number of responses to each question rather than the total number of cases. The missingness of symptom responses ranged from 2.5% (unable to play a useful part in life) to 0% (sleep badly) in Ethiopia, 13.3% (unable to play a useful part in life) to 0.8% (headaches) in Andhra Pradesh, and 6.6% (lost interest in things) to 0.3% (uncomfortable feelings in stomach) in Vietnam.

Analyses of potential correlates of distress caseness were conducted separately for each of the three countries. Univariate associations were explored using simple logistic regression. With the exception of birth weight and maternal disability, all variables were entered into a multiple linear regression model to test for collinearity using variable inflation factors (VIFs), which produced mean VIFs of 1.44, 1.26, and 1.34 for Ethiopia, Andhra Pradesh, and Vietnam, respectively, with no VIF greater than 3 indicating minimal collinearity. The multiple logistic regression models were further evaluated by stepwise removal of nonsignificant variables including years lived in the community, antenatal care, citizenship activities, and social support. All variables retained after stepwise removal for any of the three countries were included in the final multiple logistic regression models for all three countries to enable cross-country comparisons. Maternal age, child age, and child sex were included regardless of their level of association with the outcome because of their recognized relevance to maternal mental distress. Population average estimates were derived using generalized estimating equations (GEE) with robust standard errors to account for clustered sampling, which explained 3%, 22%, and 10% of the variance in Ethiopia, Andhra Pradesh, and Vietnam, respectively. The amount of correlate missing data was limited. With the exception of the social support variable, which was missing 18% total (52% in Ethiopia), all included variables had missingness of less than 5%. Multiple imputation using chained equations [48] was used to provide appropriate standard errors to account for missing data.

## 3. Results

Demographic characteristics of each of the three study populations are presented in Table 2. All three samples represent women with average age in the mid-20s (mean ranging from 24 to 27 by country) approximately 1 year since childbirth, reporting low to moderate household wealth (mean wealth index 0.21–0.44), with a considerable length of time lived (means of 9–17 years) in predominantly rural (66–80%) communities. There were statistically significant differences in proportion of the Ethiopian, Indian, and Vietnamese samples, respectively, in terms of completing primary education (21, 40, and 73%), participating in multiple economic activities (13, 19, and 71%), and experiencing economic shocks (70, 44, and 42%). There were also significant differences in proportion of unwanted pregnancies (37, 8, and 17%), lack of antenatal care (52, 11, and 24%), and death of a child (26, 11, and 6%). Based on the cutoff score on the SRQ-20 of at least 8 symptoms, prevalence of probable clinically significant maternal distress was 33% in Ethiopia, 30% in Andhra Pradesh, and 21% in Vietnam, as previously reported in Harpham et al. [37].

### 3.1. Symptom Endorsement

Mothers identified as probable cases endorsed an average of 11.4 (SD = 2.8) symptoms in Ethiopia, which was slightly higher than the 10.7 (SD = 2.3) in Andhra Pradesh and 10.6 (SD = 2.6) in Vietnam (*p* < 0.001). Mothers who were identified as* not* probable cases endorsed an average of 3.1 (SD = 2.2) symptoms in Ethiopia and 3.2 (SD = 2.1) in Andhra Pradesh, slightly higher than the 2.6 (SD = 2.2) in Vietnam (*p* < 0.001).

Figure 1 displays the prevalence for endorsing each symptom among probable cases by country. In Ethiopia, over three-quarters of cases reported being easily tired, having headaches, feeling tired all the time, and feeling unhappy (78.6, 78.3, 76.3, and 76%, resp.), while shaking hand was the least reported symptom (18.7%). In Andhra Pradesh, the most common symptoms were being easily tired and feeling unhappy (81.2 and 79.9%), whereas only a quarter of cases reported feeling like they were a worthless person (26.8%). In Vietnam, six symptoms were reported by over three-quarters of cases, feeling nervous, being easily tired, headaches, being tired all the time, being unhappy, and poor appetite (89.2, 88.9, 88.5, 79.2, 79.1, and 75.5%), while a quarter or less reported feeling worthless, feeling you just could not go on, and being unable to play a useful part in life (17.8, 21.2, and 25.1%).

Chi-square tests indicated statistically significant differences between endorsement prevalence among probable cases by country for 19 of the 20 questions at *p* ≤ 0.001; variation in a subset of symptoms appeared to be particularly visible. On three items, Vietnam differed markedly from Ethiopia and Andhra Pradesh, respectively; these included higher reporting of poor appetite (75.5%, compared to 56.8% and 49.6%), lower endorsement of feeling one could not go on (21.2%, compared to 51.8% and 50.7%), and less difficulty enjoying daily activities (35%, compared to 59.4% and 57.5%). There were also three symptoms in which the symptom prevalence in Ethiopia was notably higher relative to Andhra Pradesh and Vietnam, respectively. These included feeling unable to play a useful part in life (48.6%, compared to 31% and 25%), loss of interest in things (55.3% compared to 36.1% or 32.6%), and feeling worthless (56.2% relative to 26.8% and 17.8%). Shaking hands also appeared to be relatively uncommon in Ethiopia (18.7%) compared to Andhra Pradesh (58.8%). One item, “Do you feel unhappy?,” produced similar endorsement levels (76–80%, *X* ^2^ = 2.87, *p* = 0.238) in all three countries.

### 3.2. Sociodemographic Correlates

Table 3 presents the relative odds of maternal distress with 95% confidence intervals for each of the correlates of interest. In Ethiopia, having an unwanted pregnancy (OR = 1.92, *p* < 0.001), having more than three children (OR = 1.40, *p* = 0.010), experiencing an economic shock (OR = 1.96, *p* < 0.001), the index child having a life-threatening health event (OR = 1.86, *p* < 0.001), and having high (relative to low) group membership (OR = 1.72, *p* = 0.015) were all associated with increased odds of poor mental health. However, having high (relative to low/medium) cognitive social capital (OR = 0.57, *p* = 0.013) was associated with decreased odds of poor mental health. Marginally significant results included living in urban settings (OR = 1.73, *p* = 0.049), having no partner (1.32, *p* = 0.054), and having a child with a long-term health problem (OR = 1.46, *p* = 0.054).

In Andhra Pradesh, increasing maternal age (OR = 1.03, *p* = 0.034) and the index child experiencing a life-threatening event (OR = 1.43, *p* = 0.001) or having a long-term health problem (OR = 2.15, *p* < 0.001) were all associated with increased odds of poor mental health. However, higher household wealth (OR = 0.15, *p* < 0.001) was associated with decreased odds of poor mental health. Marginally significant findings were found for low maternal education (OR = 1.23, *p* = 0.050) and having a female index child (OR = 0.83, *p* = 0.052).

In Vietnam, being unmarried (OR = 3.52, *p* < 0.001), living in an urban setting (OR = 2.82, *p* < 0.001), experiencing an economic shock (OR = 2.34, *p* < 0.001), the index child having a life-threatening event (OR = 1.60, *p* = 0.003) or long-term health condition (OR = 3.72, *p* < 0.001), and having experienced the death of a child (OR = 1.72, *p* = 0.007) were associated with higher odds of maternal distress. However, higher household wealth (OR = 0.12, *p* < 0.001), participating in* any* livelihood activities (one: OR = 0.65, *p* = 0.003; two or more: OR = 0.58, *p* = 0.001), and having high cognitive social capital (OR = 0.48, *p* < 0.001) were all associated with lower odds of maternal distress. Marginally significant findings were found for being involved in a medium (relative to low) number of groups (OR = 1.32, *p* = 0.048).

## 4. Discussion

The current study assessed symptom endorsement by mothers meeting criteria for maternal distress and evaluated the consistency of associations between maternal distress and recognized risk factors across three LMIC settings. We identified similar patterns of symptom prevalence across the three countries. In particular, there was high and consistent reporting of feeling unhappy among probable cases of maternal distress across all three samples. While the overall similarity between countries was striking, noteworthy exceptions regarding symptom endorsement, including higher reporting of cognitive symptoms in Ethiopia and higher reporting of low energy and somatic complaints in Vietnam, were also observed.

With regard to associated risk factors, negative life events and the index child experiencing either a life-threatening event or long-term health problem were consistently associated with increased odds of distress across all three samples. The reporting of unhappiness, a hallmark feature of depression, across the three settings is consistent with major cross-national studies reporting that as many as three-quarters of respondents report sadness as a symptom of depression [49–51]. Clinically, sadness in people not currently meeting criteria for depression has been shown in the US to be an important predictor of depressive disorder onset within the following year and is critical for preventive screening [52]. The stability of this symptom in the study samples suggests the potential for similar predictive utility in these settings, although with only cross-sectional data the relative risk of onset in these samples could not be assessed.

Variation in symptom prevalence rates was identified for the items assessing the mother's feelings of utility, interest in things, and feelings of worthlessness—three items which have previously been found to load onto a single “Depressive Thoughts” factor [53]. The Ethiopian sample endorsed all three of these symptoms at higher rates than the samples from Andhra Pradesh and Vietnam. Previous qualitative research among Ethiopians has suggested that cognitive features may be viewed as* causes* of distress, with symptoms manifesting as physical complaints in the head, heart, and stomach [54]. Vietnamese women were less likely to endorse these cognitive symptoms but reported high rates for being easily tired and tired all the time, as well as having headaches and poor appetites, which have previously been shown to load on “Decreased Energy” and “Somatic” factors [53]. This conforms with clinical suggestions that low energy and sleep problems can highlight depressive moods among southeast Asian women [55]. These differences may illustrate ways in which culture influences the local expression of maternal distress and presents potential areas for further research in treatment adaptations and developing better screening tools that identify women for services as well as providing an avenue for further exploration as to how these may interact with child health outcomes. For example, perhaps mental distress expressed in terms of low energy and health complaints may involve more functional impairment than cognitive expressions, which may in turn have more adverse effects on children's healthy development.

We identified both common and distinct correlates of maternal distress across the three study samples. As previously mentioned, the most consistent findings related to negative life events and the index child experiencing a life-threatening event or long-term health problem. These factors were associated with increased odds of distress in all three settings. Higher levels of group membership also displayed a trend toward increased odds of distress across samples, which replicates a previous finding in this same sample [47] and has also been observed elsewhere [56]. As these data are cross-sectional, the causal relationship between group membership and maternal distress is unclear. Noting that support from groups was measured separately from group membership, however, one could potentially conclude that group membership itself does not offer support and may present an additional stressor for mothers. It is also noteworthy that social support itself did not explain additional variance in outcomes and was excluded from the final adjusted models; this may have been due to the higher percentage of missing data in this variable, rather than a true lack of association.

Higher wealth and cognitive social capital were generally associated with lower odds of maternal distress, consistent with prior literature [57, 58]. The higher odds of distress associated with maternal age in Andhra Pradesh were somewhat unexpected, as the women represented a rather homogenously aged group of women averaging in the mid-20s; the increased odds were minimal and may have been found by chance. Maternal distress was not associated with child age (within the range of 6–18 months), consistent with prior findings that maternal depression stabilizes after the first six months after childbirth [59]. Failure to replicate prior research findings of associations for marital status and cognitive social capital in the Indian sample is likely due to the minimal variation across these characteristics in that sample. This raises some question as to whether these factors are truly less prevalent in the population of poor mothers in Andhra Pradesh or whether the process of sentinel site surveillance does not fully represent the source population of poor mothers in that region. Likewise, the lack of association between maternal distress and wealth in Ethiopia may reflect the higher poverty level in this group as a whole rather than a true absence of impact.

A number of associations also emerged that were unique to each setting. Exclusive to Ethiopia, both having an unwanted pregnancy and having more than three children elevated risk for maternal mental distress. Unique to Andhra Pradesh, child sex (female) was marginally protective, although this runs counter to prior findings of increased risk of maternal distress associated with female children in the Indian context [60]. Vietnam was the only setting in which experiencing the death of a child remained significantly associated with mental distress after adjusting for all other factors, and having a child with a long-term health problem—while trending toward higher odds in all countries—was the strongest predictor of increased odds of mental distress in Vietnam. Lastly, participation in livelihood activities appeared to be differentially associated depending on context: in both Andhra Pradesh and Ethiopia, two or more livelihood activities in the past year trended toward increased odds of distress, whereas* any* livelihood activities in Vietnam were associated with significantly* decreased* odds of distress.

These unique results support the hypothesis that it is not absolute poverty but relative deprivation and its associated adversities within a context that may take the greatest toll on maternal mental health [58, 61]. The distressed women in Ethiopia appear to live in an environment in which poor women with low education are disempowered to make decisions about reproductive health and healthcare for themselves and their families; the result may be that large families place particular strain maternal resources. The context in India, with the lowest proportion of unplanned pregnancies, highest utilization of antenatal care, and over 99% of women in the sample married, suggests that when women have greater control over their reproductive health, economic factors such as wealth, livelihoods, and economic shocks may be more dominant influences on their wellbeing. And in Vietnam, where more women were completing basic education and participating in livelihoods, child wellbeing appears to be the domain most associated with maternal mental health. The direction of causality is unclear, but as prior literature has also documented high levels of distress among Vietnamese mothers of children with disabilities [62], it is possible that this finding reflects an impact of poor child health on maternal distress. These are all hypotheses as to the different mechanisms influencing maternal distress across these contexts, which deserve further empirical evaluation. The findings on the correlates of maternal distress should be interpreted within the historical context. As YL baseline data were collected in 2002, the relevance of identified correlates may be different for women today based on recent social and cultural developments.

One promising direction for future research involves evaluation of potential associations of maternal distress expression and correlates with child health outcomes and, in particular, how these associations may vary across settings. For example, in a four-country analysis of the YL data, Harpham et al. [37] report associations between maternal common mental disorders (CMD) and child nutritional status in India and Vietnam, but not in Peru and Ethiopia. Medhin et al. [63] also reported no significant associations between common mental disorders and child health in Ethiopia, whereas Hadley et al. [64] found strong associations in the same country. Data are still being collected on women in the YL study; thus, future studies can build on the current analysis to examine how variation in symptom prevalence and correlates may be predictive of longitudinal health and wellbeing outcomes for both the mother and child.

### 4.1. Limitations

Although the procedures of the Young Lives data collection included translation and pilot testing, the standardized YL instrument did not include potentially relevant local idioms of distress; as a result, we may have missed capturing culturally specific symptoms of distress in the three settings. Also, for these specific populations of postpartum women, the cut point of 8 or more symptoms was only explicitly validated in the Vietnam sample [46]. It is probable that some misclassification of maternal distress cases occurred, which is likely to have attenuated the strength of the observed associations.

Additionally, oversampling for women living in poverty may have produced homogenous samples not reflective of the wider experience of mothers in these three countries, such that findings are likely not generalizable to the populations of postnatal women in each country, particularly in India, where the sample was drawn from only one region of the country. It was also difficult to explore certain correlates due to small sample sizes; for example, evaluating the experiences of widowed, divorced, or never married women separately was not possible given the infrequency with which these categories were reported. The cross-sectional nature of this study also precludes inference regarding cause and effect; future studies should examine these associations across time. The analysis also did not include factors such as disability status, paternal characteristics, and intimate partner relationship stressors, which have previously been shown to be associated with maternal distress [3] and which, if included, may have impacted the adjusted estimates. Finally, due to multiple testing some observed associations may have been found by chance; given the exploratory nature of the analysis, future research to replicate these findings is warranted.

## 5. Conclusions

A strength of this study is identification of common and distinct mental health experiences of poor mothers across three diverse settings in which postnatal distress is common and has been differentially associated with negative early child health indicators. This study supports the recognized association between poverty and maternal mental distress in LMIC settings. This cycle is particularly concerning due to the potential long-term negative impact on child health of psychologically distressed mothers. Distressed mothers are less able to care for themselves and their children or to leverage social supports to do so. At the same time, the stress of mothering a child with poor health can also increase the risk of maternal distress. A 15-year longitudinal study of childhood poverty in Ethiopia, Andhra Pradesh, Peru, and Vietnam is exploring questions of this nature. Baseline data indicated the association between these factors may be mediated by the culture in which the women live [37]. The current analysis supports this conclusion.

This study identified country-specific and unique patterns of symptom prevalence and sociodemographic correlates in the Ethiopia, Andhra Pradesh, and Vietnam samples. These results contribute to the literature on maternal mental health by identifying similarities and differences in symptom presentation with implications for clinical practice, as well as potential areas of divergence in the experience of maternal distress across LMIC contexts. Findings suggest that “feeling unhappy” is a consistent experience of maternal distress across cultures and may be leveraged for early screening and prevention. However, variation in symptom prevalence rates between settings was also notable. These findings highlight the value of further exploration of potential cross-cultural differences in experiences of maternal mental distress.

## Figures and Tables

**Table 1:** Potential maternal distress correlates considered in analysis.

Maternal	Pregnancy/child	Household
Age Marital status Education Disability status^1^ Livelihood activities Cognitive social capital Group membership Social support^2^ Citizenship activities^2^ Years lived in community^2^	Unwanted pregnancy Antenatal care^2^ Child age Child sex Long-term health problem Life-threatening incident Birth weight^1^	Wealth index Urban/rural setting Economic shock More than 3 children Death of a child

^1^Excluded from initial multiple regression models.

^2^Excluded from final models after stepwise deletion.

**Table 2:** Sample demographics by country.

	Ethiopia	India	Vietnam	*p* value^*∗*^
	(*n* = 1906)	(*n* = 1886)	(*n* = 1855)	
Household wealth index (mean, SD)	0.21 (.17)	0.41 (.20)	0.44 (2.23)	<0.001
Maternal age in years (mean, SD)	27.4 (6.3)	23.7 (4.3)	27.2 (5.8)	<0.001
Years lived in community (mean, SD)	15.5 (10.6)	9.1 (7.7)	17.4 (11.2)	<0.001
Age of child in months (mean, SD)	11.6 (3.6)	11.7 (3.5)	11.6 (3.2)	0.122
Number of children (mean, SD)	3.6 (2.4)	2.0 (1.2)	1.9 (1.2)	<0.001
Index child is female (%)	47.01	45.92	48.41	0.310
Marital status (%)				<0.001
Married	86.62	99.42	97.68	
Divorced	7.92	0.32	1.08	
Single	3.52	0.11	1.02	
Widowed	1.94	0.16	0.22	
Urban setting (%)	33.95	25.5	19.95	<0.001
Education level (%)				<0.001
Completed primary	21.14	40.40	72.83	
Did not complete primary	73.19	59.49	27.17	
Livelihood activities in past year (%)				<0.001
None	42.81	50.37	6.74	
One	43.70	30.65	22.16	
Two or more	13.48	18.98	71.11	
Unwanted pregnancy (%)	37.15	8.01	17.20	<0.001
Experienced economic shock (%)	70.41	43.80	42.37	<0.001
Child life-threatening incident (%)	30.43	22.59	12.88	<0.001
Child long-term health problem (%)	10.02	4.40	4.26	<0.001
Death of a child (%)	26.50	11.13	5.82	<0.001
Group membership (%)				<0.001
Low (0)	23.03	70.78	73.26	
Medium (1-2)	55.93	28.58	24.69	
High (3 or more)	12.01	0.32	2.05	
Cognitive social capital (%)				<0.001
Low/medium	11.23	5.14	9.38	
High	87.46	92.52	90.40	
% meeting distress criteria (i.e., ≥8 symptoms)	33.0	30.1	21.2	<0.001

^*∗*^Comparisons using chi-square for categorical variables, ANOVA for continuous variables.

**Table 3:** Correlates of maternal distress.

	Ethiopia		Andhra Pradesh		Vietnam	
	OR	95% CI	OR	95% CI	OR	95% CI
Maternal age (years)	1.01	[0.99, 1.03]	1.03^*∗*^	[1.00, 1.06]	1.00	[0.97, 1.03]
Child age (months)	1.00	[0.97, 1.04]	1.01	[0.98, 1.04]	1.01	[0.98, 1.05]
Female (versus male) child	0.94	[0.77, 1.15]	0.83	[0.70, 1.00]	0.96	[0.82, 1.13]
Household wealth index	0.47	[0.10, 2.11]	0.15^*∗∗*^	[0.07, 0.34]	0.12^*∗∗*^	[0.06, 0.23]
Urban (versus rural) setting	1.73^*∗*^	[1.00, 2.98]	1.56	[0.50, 4.89]	2.82^*∗∗*^	[1.71, 4.64]
No partner (versus having partner)	1.32	[0.99, 1.74]	2.29	[0.90, 5.79]	3.52^*∗∗*^	[1.98, 6.24]
Low (versus more than primary) education	1.15	[0.79, 1.69]	1.23	[1.00, 1.51]	1.04	[0.82, 1.33]
Unwanted (versus wanted) pregnancy	1.92^*∗∗*^	[1.62, 2.27]	1.07	[0.61, 1.88]	0.81	[0.55, 1.20]
Having > 3 (versus 1–3) children	1.40^*∗*^	[1.08, 1.81]	1.06	[0.60, 1.89]	1.18	[0.82, 1.69]
Experiencing an economic shock (versus not)	1.96^*∗∗*^	[1.43, 2.69]	1.29	[0.97, 1.73]	2.34^*∗∗*^	[1.73, 3.16]
Child life-threatening incident (versus not)	1.86^*∗∗*^	[1.51, 2.28]	1.43^*∗∗*^	[1.16, 1.77]	1.60^*∗∗*^	[1.17, 2.19]
Child long-term health problem (versus not)	1.46	[0.99, 2.16]	2.15^*∗∗*^	[1.50, 3.09]	3.72^*∗∗*^	[2.31, 6.01]
Death of a child (versus not)	1.12	[0.87, 1.43]	1.11	[0.82, 1.51]	1.73^*∗∗*^	[1.16, 2.57]
Maternal livelihood activities (ref: none)						
One	1.02	[0.77, 1.35]	1.11	[0.83, 1.50]	0.65^*∗∗*^	[0.48, 0.86]
Two or more	1.37	[0.94, 1.98]	1.17	[0.80, 1.70]	0.58^*∗∗*^	[0.42, 0.79]
Maternal group membership (ref: low)						
Med	0.94	[0.70, 1.24]	1.16	[0.95, 1.42]	1.32^*∗*^	[1.00, 1.74]
High	1.72^*∗*^	[1.11, 2.68]	0.61	[0.11, 3.40]	1.18	[0.59, 2.38]
High (versus low/medium) CSC	0.57^*∗*^	[0.37, 0.89]	0.67	[0.42, 1.07]	0.48^*∗∗*^	[0.36, 0.64]

^*∗*^ *p* < 0.05.

^*∗∗*^ *p* < 0.01.

Population averages from multiple logistic regression using GEE estimation with robust standard errors.

## Abbreviations

YLD: Years lost due to disability
LMIC: Low and middle income country
UNDP: United Nations Development Program
CMD: Common mental disorders
SRQ-20: Self-Reporting Questionnaire-20 Items.
